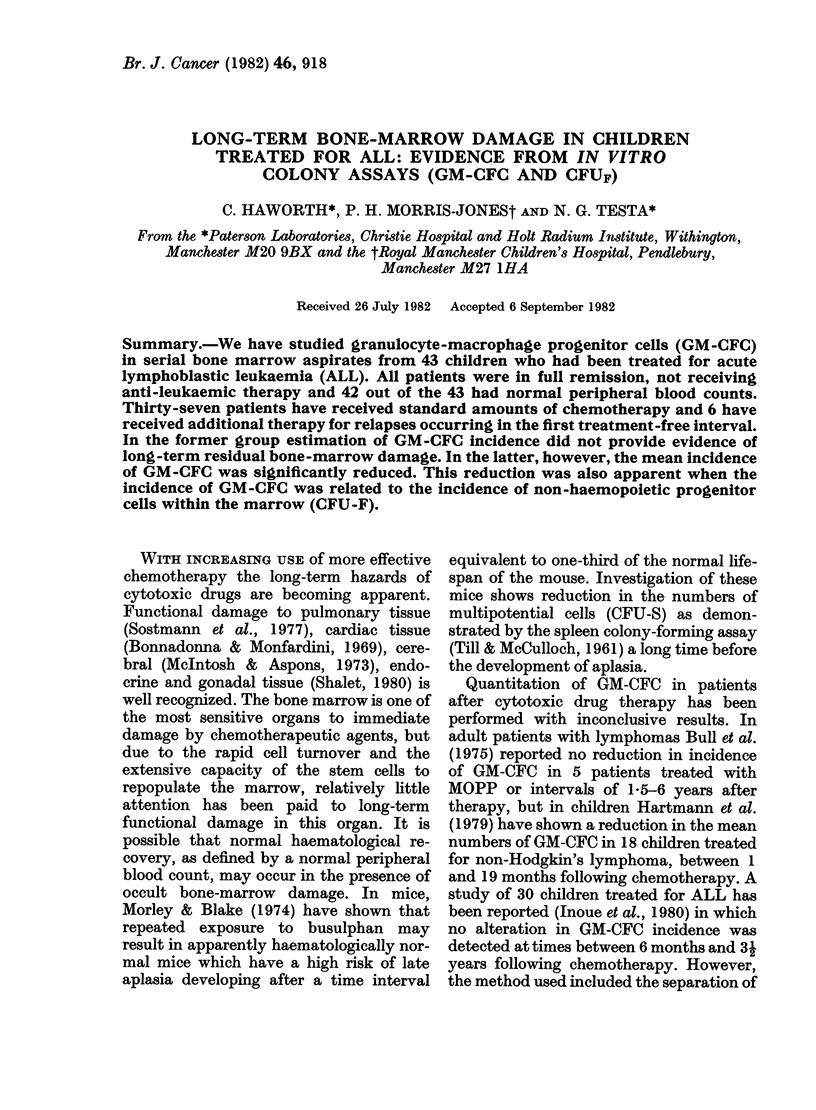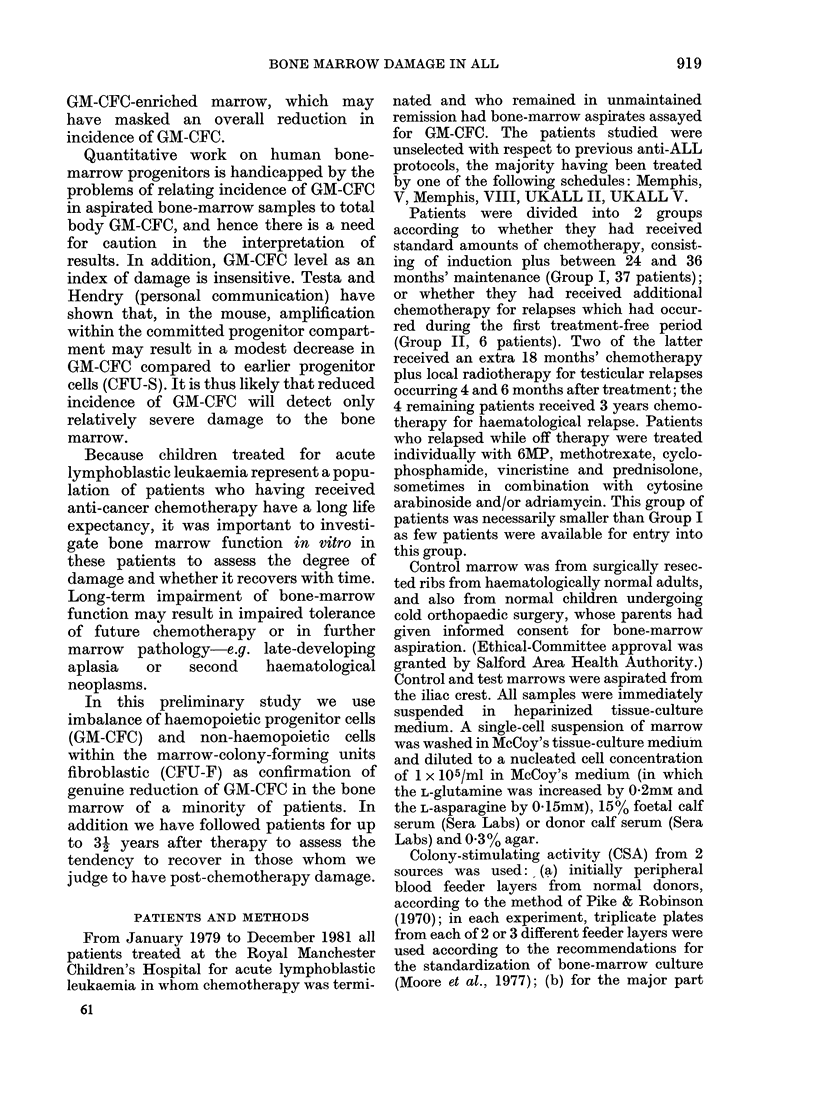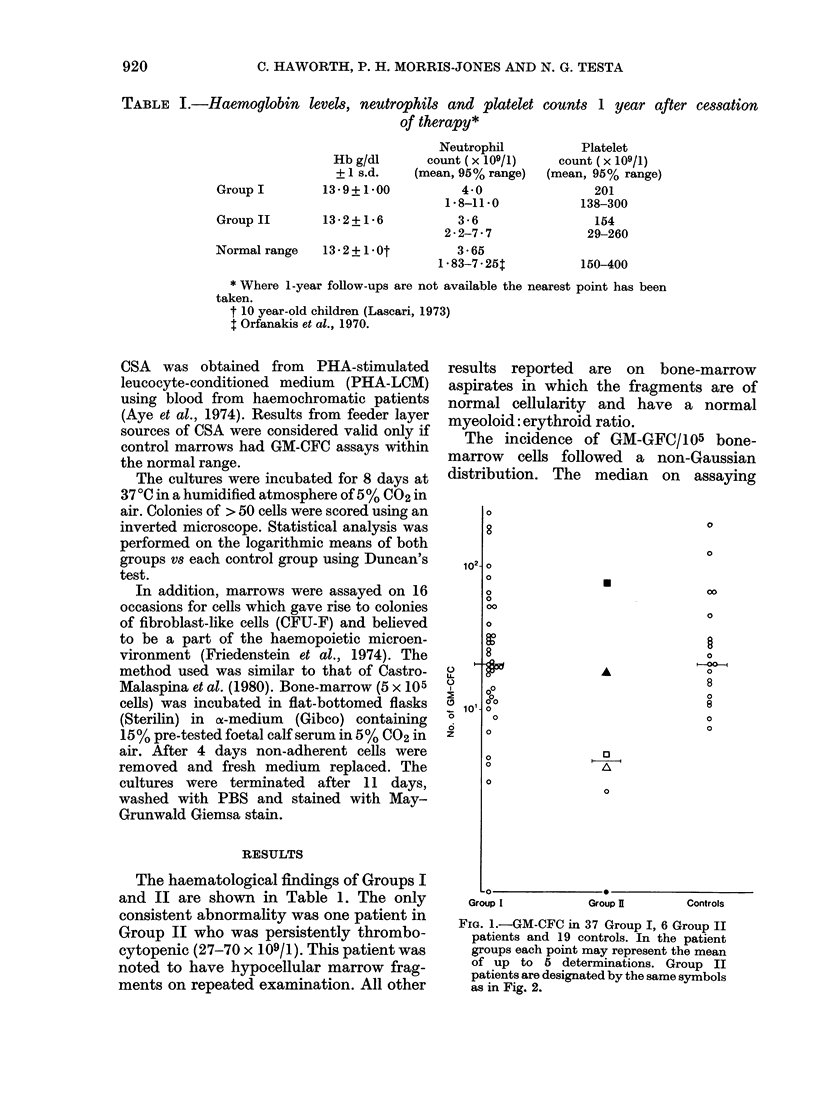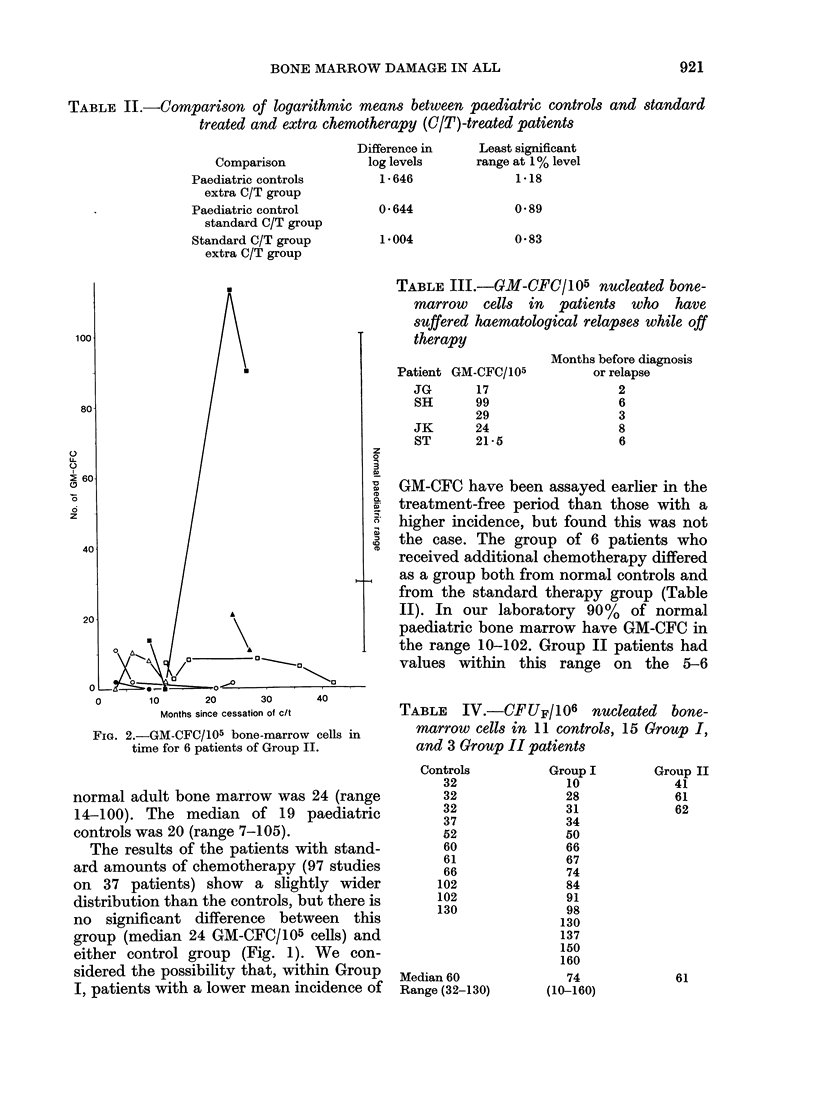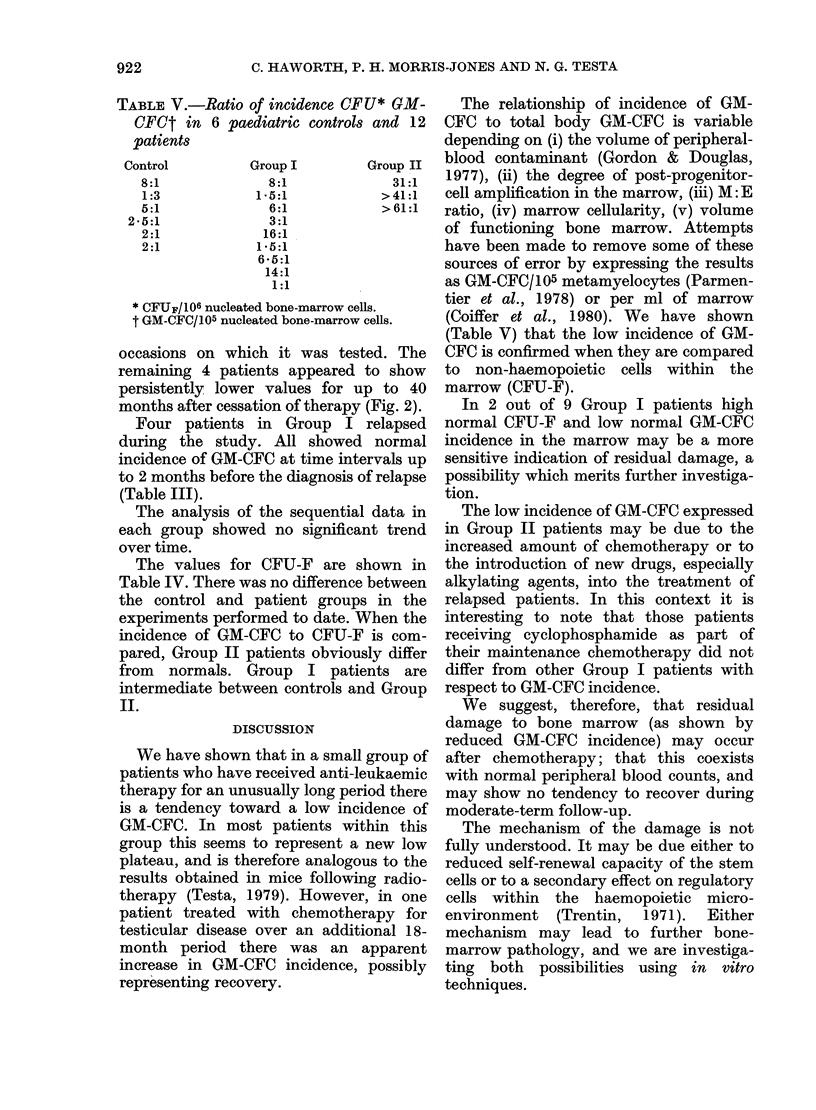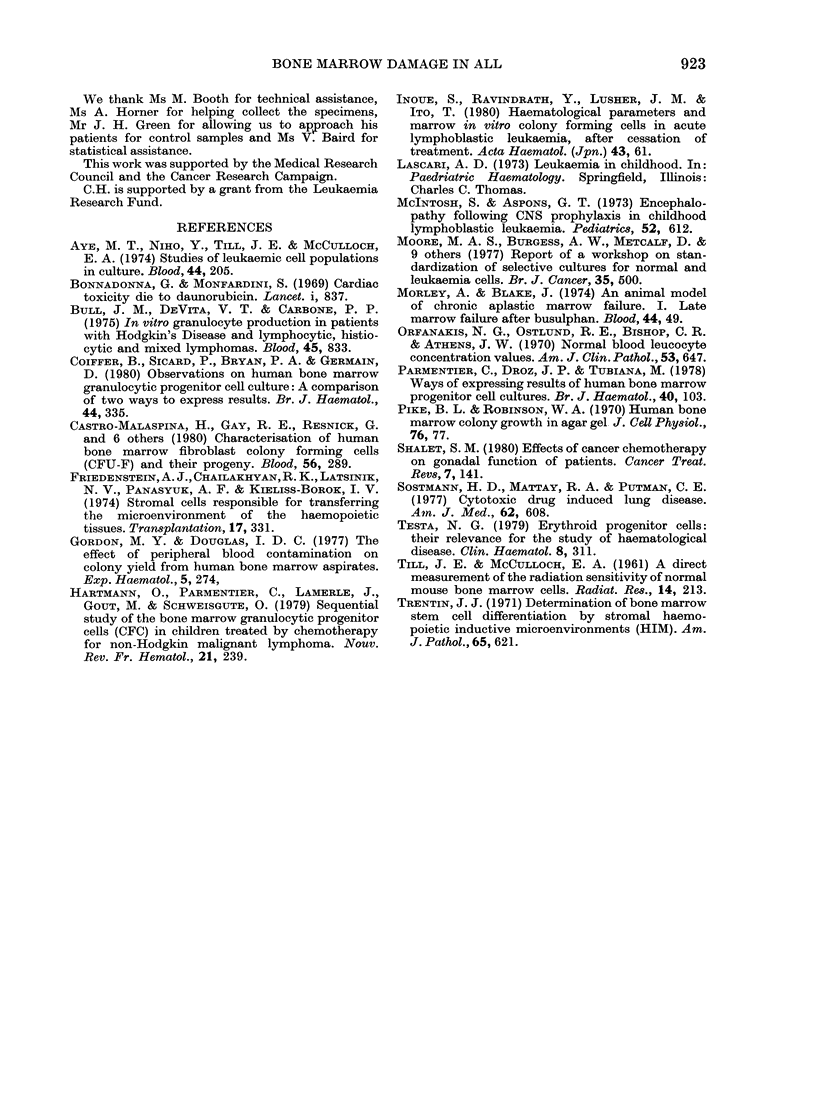# Long-term bone-marrow damage in children treated for ALL: evidence from in vitro colony assays (GM-CFC and CFUF).

**DOI:** 10.1038/bjc.1982.302

**Published:** 1982-12

**Authors:** C. Haworth, P. H. Morris-Jones, N. G. Testa

## Abstract

We have studied granulocyte-macrophage progenitor cells (GM-CFC) in serial bone marrow aspirates from 43 children who had been treated for acute lymphoblastic leukaemia (ALL). All patients were in full remission, not receiving anti-leukaemic therapy and 42 out of the 43 had normal peripheral blood counts. Thirty-seven patients have received standard amounts of chemotherapy and 6 have received additional therapy for relapses occurring in the first treatment-free interval. In the former group estimation of GM-CFC incidence did not provide evidence of long-term residual bone-marrow damage. In the latter, however, the mean incidence of GM-CFC was significantly reduced. This reduction was also apparent when the incidence of GM-CFC was related to the incidence of non-haemopoietic progenitor cells within the marrow (CFU-F).


*Br. J. Cancer* (1982) **46**, 918

### LONG-TERM BONE-MARROW DAMAGE IN CHILDREN TREATED FOR ALL: EVIDENCE FROM *IN VITRO* COLONY ASSAYS (GM-CFC AND $CFU_F$)

C. HAWORTH*, P. H. MORRIS-JONES† AND N. G. TESTA*

*From the *Paterson Laboratories, Christie Hospital and Holt Radium Institute, Withington, Manchester M20 9BX and the †Royal Manchester Children's Hospital, Pendlebury, Manchester M27 1HA*

Received 26 July 1982 Accepted 6 September 1982

**Summary.—We have studied granulocyte-macrophage progenitor cells (GM-CFC) in serial bone marrow aspirates from 43 children who had been treated for acute lymphoblastic leukaemia (ALL). All patients were in full remission, not receiving anti-leukaemic therapy and 42 out of the 43 had normal peripheral blood counts. Thirty-seven patients have received standard amounts of chemotherapy and 6 have received additional therapy for relapses occurring in the first treatment-free interval. In the former group estimation of GM-CFC incidence did not provide evidence of long-term residual bone-marrow damage. In the latter, however, the mean incidence of GM-CFC was significantly reduced. This reduction was also apparent when the incidence of GM-CFC was related to the incidence of non-haemopoietic progenitor cells within the marrow (CFU-F).**

WITH INCREASING USE of more effective chemotherapy the long-term hazards of cytotoxic drugs are becoming apparent. Functional damage to pulmonary tissue (Sostmann *et al.*, 1977), cardiac tissue (Bonnadonna & Monfardini, 1969), cerebral (McIntosh & Aspons, 1973), endocrine and gonadal tissue (Shalet, 1980) is well recognized. The bone marrow is one of the most sensitive organs to immediate damage by chemotherapeutic agents, but due to the rapid cell turnover and the extensive capacity of the stem cells to repopulate the marrow, relatively little attention has been paid to long-term functional damage in this organ. It is possible that normal haematological recovery, as defined by a normal peripheral blood count, may occur in the presence of occult bone-marrow damage. In mice, Morley & Blake (1974) have shown that repeated exposure to busulphan may result in apparently haematologically normal mice which have a high risk of late aplasia developing after a time interval equivalent to one-third of the normal life-span of the mouse. Investigation of these mice shows reduction in the numbers of multipotential cells (CFU-S) as demonstrated by the spleen colony-forming assay (Till & McCulloch, 1961) a long time before the development of aplasia.

Quantitation of GM-CFC in patients after cytotoxic drug therapy has been performed with inconclusive results. In adult patients with lymphomas Bull *et al.* (1975) reported no reduction in incidence of GM-CFC in 5 patients treated with MOPP or intervals of 1·5–6 years after therapy, but in children Hartmann *et al.* (1979) have shown a reduction in the mean numbers of GM-CFC in 18 children treated for non-Hodgkin's lymphoma, between 1 and 19 months following chemotherapy. A study of 30 children treated for ALL has been reported (Inoue *et al.*, 1980) in which no alteration in GM-CFC incidence was detected at times between 6 months and 3½ years following chemotherapy. However, the method used included the separation of

GM-CFC-enriched marrow, which may have masked an overall reduction in incidence of GM-CFC.

Quantitative work on human bone-marrow progenitors is handicapped by the problems of relating incidence of GM-CFC in aspirated bone-marrow samples to total body GM-CFC, and hence there is a need for caution in the interpretation of results. In addition, GM-CFC level as an index of damage is insensitive. Testa and Hendry (personal communication) have shown that, in the mouse, amplification within the committed progenitor compartment may result in a modest decrease in GM-CFC compared to earlier progenitor cells (CFU-S). It is thus likely that reduced incidence of GM-CFC will detect only relatively severe damage to the bone marrow.

Because children treated for acute lymphoblastic leukaemia represent a population of patients who having received anti-cancer chemotherapy have a long life expectancy, it was important to investigate bone marrow function *in vitro* in these patients to assess the degree of damage and whether it recovers with time. Long-term impairment of bone-marrow function may result in impaired tolerance of future chemotherapy or in further marrow pathology—*e.g.* late-developing aplasia or second haematological neoplasms.

In this preliminary study we use imbalance of haemopoietic progenitor cells (GM-CFC) and non-haemopoietic cells within the marrow-colony-forming units fibroblastic (CFU-F) as confirmation of genuine reduction of GM-CFC in the bone marrow of a minority of patients. In addition we have followed patients for up to 3½ years after therapy to assess the tendency to recover in those whom we judge to have post-chemotherapy damage.

### PATIENTS AND METHODS

From January 1979 to December 1981 all patients treated at the Royal Manchester Children's Hospital for acute lymphoblastic leukaemia in whom chemotherapy was terminated and who remained in unmaintained remission had bone-marrow aspirates assayed for GM-CFC. The patients studied were unselected with respect to previous anti-ALL protocols, the majority having been treated by one of the following schedules: Memphis, V, Memphis, VIII, UKALL II, UKALL V.

Patients were divided into 2 groups according to whether they had received standard amounts of chemotherapy, consisting of induction plus between 24 and 36 months' maintenance (Group I, 37 patients); or whether they had received additional chemotherapy for relapses which had occurred during the first treatment-free period (Group II, 6 patients). Two of the latter received an extra 18 months' chemotherapy plus local radiotherapy for testicular relapses occurring 4 and 6 months after treatment; the 4 remaining patients received 3 years chemotherapy for haematological relapse. Patients who relapsed while off therapy were treated individually with 6MP, methotrexate, cyclophosphamide, vincristine and prednisolone, sometimes in combination with cytosine arabinoside and/or adriamycin. This group of patients was necessarily smaller than Group I as few patients were available for entry into this group.

Control marrow was from surgically resected ribs from haematologically normal adults, and also from normal children undergoing cold orthopaedic surgery, whose parents had given informed consent for bone-marrow aspiration. (Ethical-Committee approval was granted by Salford Area Health Authority.) Control and test marrows were aspirated from the iliac crest. All samples were immediately suspended in heparinized tissue-culture medium. A single-cell suspension of marrow was washed in McCoy's tissue-culture medium and diluted to a nucleated cell concentration of $1 \times 10^5$/ml in McCoy's medium (in which the L-glutamine was increased by 0·2mM and the L-asparagine by 0·15mM), 15% foetal calf serum (Sera Labs) or donor calf serum (Sera Labs) and 0·3% agar.

Colony-stimulating activity (CSA) from 2 sources was used: (a) initially peripheral blood feeder layers from normal donors, according to the method of Pike & Robinson (1970); in each experiment, triplicate plates from each of 2 or 3 different feeder layers were used according to the recommendations for the standardization of bone-marrow culture (Moore *et al.*, 1977); (b) for the major part

TABLE I.—*Haemoglobin levels, neutrophils and platelet counts* 1 *year after cessation of therapy**

| | Hb g/dl ±1 s.d. | Neutrophil count (× $10^9$/l) (mean, 95% range) | Platelet count (× $10^9$/l) (mean, 95% range) |
|---|---|---|---|
| Group I | 13·9±1·00 | 4·0<br>1·8–11·0 | 201<br>138–300 |
| Group II | 13·2±1·6 | 3·6<br>2·2–7·7 | 154<br>29–260 |
| Normal range | 13·2±1·0† | 3·65<br>1·83–7·25‡ | 150–400 |

* Where 1-year follow-ups are not available the nearest point has been taken.
† 10 year-old children (Lascari, 1973)
‡ Orfanakis *et al.*, 1970.

CSA was obtained from PHA-stimulated leucocyte-conditioned medium (PHA-LCM) using blood from haemochromatic patients (Aye *et al.*, 1974). Results from feeder layer sources of CSA were considered valid only if control marrows had GM-CFC assays within the normal range.

The cultures were incubated for 8 days at 37 °C in a humidified atmosphere of 5% $CO_2$ in air. Colonies of > 50 cells were scored using an inverted microscope. Statistical analysis was performed on the logarithmic means of both groups *vs* each control group using Duncan's test.

In addition, marrows were assayed on 16 occasions for cells which gave rise to colonies of fibroblast-like cells (CFU-F) and believed to be a part of the haemopoietic microenvironment (Friedenstein *et al.*, 1974). The method used was similar to that of Castro-Malaspina *et al.* (1980). Bone-marrow ($5 \times 10^5$ cells) was incubated in flat-bottomed flasks (Sterilin) in α-medium (Gibco) containing 15% pre-tested foetal calf serum in 5% $CO_2$ in air. After 4 days non-adherent cells were removed and fresh medium replaced. The cultures were terminated after 11 days, washed with PBS and stained with May-Grunwald Giemsa stain.

### RESULTS

The haematological findings of Groups I and II are shown in Table 1. The only consistent abnormality was one patient in Group II who was persistently thrombocytopenic (27–70 × $10^9$/l). This patient was noted to have hypocellular marrow fragments on repeated examination. All other results reported are on bone-marrow aspirates in which the fragments are of normal cellularity and have a normal myeoloid:erythroid ratio.

The incidence of GM-GFC/$10^5$ bone-marrow cells followed a non-Gaussian distribution. The median on assaying

FIG. 1.—GM-CFC in 37 Group I, 6 Group II patients and 19 controls. In the patient groups each point may represent the mean of up to 5 determinations. Group II patients are designated by the same symbols as in Fig. 2.

TABLE II.—*Comparison of logarithmic means between paediatric controls and standard treated and extra chemotherapy (C/T)-treated patients*

| Comparison | Difference in log levels | Least significant range at 1% level |
|---|---|---|
| Paediatric controls extra C/T group | 1·646 | 1·18 |
| Paediatric control standard C/T group | 0·644 | 0·89 |
| Standard C/T group extra C/T group | 1·004 | 0·83 |

FIG. 2.—GM-CFC/$10^5$ bone-marrow cells in time for 6 patients of Group II.

normal adult bone marrow was 24 (range 14–100). The median of 19 paediatric controls was 20 (range 7–105).

The results of the patients with standard amounts of chemotherapy (97 studies on 37 patients) show a slightly wider distribution than the controls, but there is no significant difference between this group (median 24 GM-CFC/$10^5$ cells) and either control group (Fig. 1). We considered the possibility that, within Group I, patients with a lower mean incidence of GM-CFC have been assayed earlier in the treatment-free period than those with a higher incidence, but found this was not the case. The group of 6 patients who received additional chemotherapy differed as a group both from normal controls and from the standard therapy group (Table II). In our laboratory 90% of normal paediatric bone marrow have GM-CFC in the range 10–102. Group II patients had values within this range on the 5–6

TABLE III.—*GM-CFC/$10^5$ nucleated bone-marrow cells in patients who have suffered haematological relapses while off therapy*

| Patient | GM-CFC/$10^5$ | Months before diagnosis or relapse |
|---|---|---|
| JG | 17 | 2 |
| SH | 99 | 6 |
| | 29 | 3 |
| JK | 24 | 8 |
| ST | 21·5 | 6 |

TABLE IV.—*$CFU_F/10^6$ nucleated bone-marrow cells in 11 controls, 15 Group I, and 3 Group II patients*

| Controls | Group I | Group II |
|---|---|---|
| 32 | 10 | 41 |
| 32 | 28 | 61 |
| 32 | 31 | 62 |
| 37 | 34 | |
| 52 | 50 | |
| 60 | 66 | |
| 61 | 67 | |
| 66 | 74 | |
| 102 | 84 | |
| 102 | 91 | |
| 130 | 98 | |
| | 130 | |
| | 137 | |
| | 150 | |
| | 160 | |
| Median 60 | 74 | 61 |
| Range (32–130) | (10–160) | |

TABLE V.—*Ratio of incidence CFU* GM-CFC† in 6 paediatric controls and 12 patients*

| Control | Group I | Group II |
|---|---|---|
| 8:1 | 8:1 | 31:1 |
| 1:3 | 1·5:1 | >41:1 |
| 5:1 | 6:1 | >61:1 |
| 2·5:1 | 3:1 | |
| 2:1 | 16:1 | |
| 2:1 | 1·5:1 | |
| | 6·5:1 | |
| | 14:1 | |
| | 1:1 | |

* $CFU_F/10^6$ nucleated bone-marrow cells.
† GM-CFC/$10^5$ nucleated bone-marrow cells.

occasions on which it was tested. The remaining 4 patients appeared to show persistently lower values for up to 40 months after cessation of therapy (Fig. 2).

Four patients in Group I relapsed during the study. All showed normal incidence of GM-CFC at time intervals up to 2 months before the diagnosis of relapse (Table III).

The analysis of the sequential data in each group showed no significant trend over time.

The values for CFU-F are shown in Table IV. There was no difference between the control and patient groups in the experiments performed to date. When the incidence of GM-CFC to CFU-F is compared, Group II patients obviously differ from normals. Group I patients are intermediate between controls and Group II.

### DISCUSSION

We have shown that in a small group of patients who have received anti-leukaemic therapy for an unusually long period there is a tendency toward a low incidence of GM-CFC. In most patients within this group this seems to represent a new low plateau, and is therefore analogous to the results obtained in mice following radiotherapy (Testa, 1979). However, in one patient treated with chemotherapy for testicular disease over an additional 18-month period there was an apparent increase in GM-CFC incidence, possibly representing recovery.

The relationship of incidence of GM-CFC to total body GM-CFC is variable depending on (i) the volume of peripheral-blood contaminant (Gordon & Douglas, 1977), (ii) the degree of post-progenitor-cell amplification in the marrow, (iii) M:E ratio, (iv) marrow cellularity, (v) volume of functioning bone marrow. Attempts have been made to remove some of these sources of error by expressing the results as GM-CFC/$10^5$ metamyelocytes (Parmentier *et al.*, 1978) or per ml of marrow (Coiffer *et al.*, 1980). We have shown (Table V) that the low incidence of GM-CFC is confirmed when they are compared to non-haemopoietic cells within the marrow (CFU-F).

In 2 out of 9 Group I patients high normal CFU-F and low normal GM-CFC incidence in the marrow may be a more sensitive indication of residual damage, a possibility which merits further investigation.

The low incidence of GM-CFC expressed in Group II patients may be due to the increased amount of chemotherapy or to the introduction of new drugs, especially alkylating agents, into the treatment of relapsed patients. In this context it is interesting to note that those patients receiving cyclophosphamide as part of their maintenance chemotherapy did not differ from other Group I patients with respect to GM-CFC incidence.

We suggest, therefore, that residual damage to bone marrow (as shown by reduced GM-CFC incidence) may occur after chemotherapy; that this coexists with normal peripheral blood counts, and may show no tendency to recover during moderate-term follow-up.

The mechanism of the damage is not fully understood. It may be due either to reduced self-renewal capacity of the stem cells or to a secondary effect on regulatory cells within the haemopoietic micro-environment (Trentin, 1971). Either mechanism may lead to further bone-marrow pathology, and we are investigating both possibilities using *in vitro* techniques.

We thank Ms M. Booth for technical assistance, Ms A. Horner for helping collect the specimens, Mr J. H. Green for allowing us to approach his patients for control samples and Ms V. Baird for statistical assistance.

This work was supported by the Medical Research Council and the Cancer Research Campaign.

C.H. is supported by a grant from the Leukaemia Research Fund.

### REFERENCES

AYE, M. T., NIHO, Y., TILL, J. E. & MCCULLOCH, E. A. (1974) Studies of leukaemic cell populations in culture. *Blood*, **44**, 205.

BONNADONNA, G. & MONFARDINI, S. (1969) Cardiac toxicity die to daunorubicin. *Lancet*. i, 837.

BULL, J. M., DEVITA, V. T. & CARBONE, P. P. (1975) *In vitro* granulocyte production in patients with Hodgkin's Disease and lymphocytic, histiocytic and mixed lymphomas. *Blood*, **45**, 833.

COIFFER, B., SICARD, P., BRYAN, P. A. & GERMAIN, D. (1980) Observations on human bone marrow granulocytic progenitor cell culture: A comparison of two ways to express results. *Br. J. Haematol.*, **44**, 335.

CASTRO-MALASPINA, H., GAY, R. E., RESNICK, G. and 6 others (1980) Characterisation of human bone marrow fibroblast colony forming cells (CFU-F) and their progeny. *Blood*, **56**, 289.

FRIEDENSTEIN, A. J., CHAILAKHYAN, R. K., LATSINIK, N. V., PANASYUK, A. F. & KIELISS-BOROK, I. V. (1974) Stromal cells responsible for transferring the microenvironment of the haemopoietic tissues. *Transplantation*, **17**, 331.

GORDON, M. Y. & DOUGLAS, I. D. C. (1977) The effect of peripheral blood contamination on colony yield from human bone marrow aspirates. *Exp. Haematol.*, **5**, 274,

HARTMANN, O., PARMENTIER, C., LAMERLE, J., GOUT, M. & SCHWEISGUTE, O. (1979) Sequential study of the bone marrow granulocytic progenitor cells (CFC) in children treated by chemotherapy for non-Hodgkin malignant lymphoma. *Nouv. Rev. Fr. Hematol.*, **21**, 239.

INOUE, S., RAVINDRATH, Y., LUSHER, J. M. & ITO, T. (1980) Haematological parameters and marrow *in vitro* colony forming cells in acute lymphoblastic leukaemia, after cessation of treatment. *Acta Haematol.* (*Jpn.*) **43**, 61.

LASCARI, A. D. (1973) Leukaemia in childhood. In: *Paedriatric Haematology*. Springfield, Illinois: Charles C. Thomas.

MCINTOSH, S. & ASPONS, G. T. (1973) Encephalopathy following CNS prophylaxis in childhood lymphoblastic leukaemia. *Pediatrics*, **52**, 612.

MOORE, M. A. S., BURGESS, A. W., METCALF, D. & 9 others (1977) Report of a workshop on standardization of selective cultures for normal and leukaemia cells. *Br. J. Cancer*, **35**, 500.

MORLEY, A. & BLAKE, J. (1974) An animal model of chronic aplastic marrow failure. I. Late marrow failure after busulphan. *Blood*, **44**, 49.

ORFANAKIS, N. G., OSTLUND, R. E., BISHOP, C. R. & ATHENS, J. W. (1970) Normal blood leucocyte concentration values. *Am. J. Clin. Pathol.*, **53**, 647.

PARMENTIER, C., DROZ, J. P. & TUBIANA, M. (1978) Ways of expressing results of human bone marrow progenitor cell cultures. *Br. J. Haematol.*, **40**, 103.

PIKE, B. L. & ROBINSON, W. A. (1970) Human bone marrow colony growth in agar gel. *J. Cell Physiol.*, **76**, 77.

SHALET, S. M. (1980) Effects of cancer chemotherapy on gonadal function of patients. *Cancer Treat. Revs*, **7**, 141.

SOSTMANN, H. D., MATTAY, R. A. & PUTMAN, C. E. (1977) Cytotoxic drug induced lung disease. *Am. J. Med.*, **62**, 608.

TESTA, N. G. (1979) Erythroid progenitor cells: their relevance for the study of haematological disease. *Clin. Haematol.* **8**, 311.

TILL, J. E. & MCCULLOCH, E. A. (1961) A direct measurement of the radiation sensitivity of normal mouse bone marrow cells. *Radiat. Res.*, **14**, 213.

TRENTIN, J. J. (1971) Determination of bone marrow stem cell differentiation by stromal haemopoietic inductive microenvironments (HIM). *Am. J. Pathol.*, **65**, 621.